# Innovations in Pancreatic Surgery

**DOI:** 10.1155/2014/963416

**Published:** 2014-07-21

**Authors:** Masahiko Hirota, Juhani Sand, Ralf Segersvärd, Roberto Cirocchi

**Affiliations:** ^1^Department of Surgery, Kumamoto Regional Medical Center, Kumamoto 860-0811, Japan; ^2^Division of Surgery, Gastroenterology and Oncology, Tampere University Hospital, P.O. Box 2000, 33521 Tampere, Finland; ^3^Division of Surgery, CLINTEC, Karolinska Institutet at Center for Digestive Diseases, Karolinska University Hospital, 141 86 Stockholm, Sweden; ^4^Department of Surgical and Biomedical Science, University of Perugia, 05100 Terni, Italy

New knowledge about the nature of pancreatic diseases and the ability to perform more complex pancreatic surgical procedures have changed the indication for surgical treatment and permitted an introduction of organ-preserving surgical techniques. The rapid development in technological innovations has improved surgeons' techniques, and accumulation of surgeons' experiences improved the outcome after pancreatic surgery.

Pancreatic cancer is one of the most devastating diseases known to mankind with the worst 5-year survival rate among neoplasms. Many surgeons created their own devices to improve the prognosis of such dismal disease, for example, no-touch approach, artery-first approach, or extensive intraoperative peritoneal lavage. By their endeavor, some lines of hope are being reported in several academic meetings.

On the other hand, recent progress in diagnostic imaging procedures enables us to find various pancreatic lesions. Among them, slow growing tumors, such as intraductal papillary mucinous neoplasm (IPMN), mucinous and serous cystic neoplasms, solid pseudopapillary tumor, and pancreatic neuroendocrine tumor, are included. The presence of these rather low malignant tumors facilitated us to devise organ-preserving minimally invasive surgery, such as laparoscopic or robotically assisted pancreatectomy. Indeed, minimally invasive surgery has become widely accepted as a superior alternative to conventional open surgery for selected patients within the field of pancreatology.

The first paper of this special issue addresses current and future intraoperative imaging modalities and their potential for improved tumor demarcation during pancreatic surgery.

The second paper presents a new surgical technique of proximal subtotal pancreatectomy with splenic artery and vein resection; the so-called pancreaticoduodenectomy with splenic artery resection (PD-SAR). PD-SAR with preoperative chemoradiotherapy seems to be a promising surgical strategy for pancreatic ductal adenocarcinoma of head and/or body with invasion of the splenic artery, with regard to the balance between operative radicality and postoperative QOL.

The third paper is on the Finnish binding (purse-string) pancreaticojejunal anastomosis (FBPJ), which was shown to reduce the risk for postoperative pancreatic fistula (POPF) after PD. In this paper, the efficacy of FBPJ after left pancreatectomy is discussed showing that FBPJ was not technically achievable in 72% of the cases and did not reduce the risk for POPF compared to the conventional hand-sewn closure. Therefore, FBPJ cannot be recommended for the routine closure of the pancreatic remnant after left pancreatectomy.

The fourth paper describes paraduodenal pancreatitis (PP) which was proposed as a synonym for duodenal dystrophy (DD) and groove pancreatitis. Although conventional PD is the main surgical option for treatment of PP today, early diagnosis makes pancreas-preserving duodenal resection (PPDR) the treatment of choice for PP. Efficacy of PPDR provides proof that PP is an entity of the duodenum and not of paraduodenal origin.

The fifth paper addresses the efficacy of combined endoscopic lithotomy (EL) plus extracorporeal shock wave lithotripsy (ESWL) and additional electrohydraulic lithotripsy (EHL) on pancreatic lithiasis. Combined EL plus ESWL therapy is regarded as the first treatment option. However, in cases where the combined therapy was not successful for stone clearance, SpyGlass guided EHL or X-ray guided EHL was effective.

The sixth paper is a systematic review of randomized controlled trials (RCTs) dealing with surgical techniques in distal pancreatectomy. Management of the pancreatic remnant after distal pancreatectomy is still a matter of debate. New well designed and carefully conducted RCTs must be performed to establish the optimal strategy for pancreatic remnant management after distal pancreatectomy.

The seventh paper presents the state of the art of pancreatic robotic surgery. With the current lack of evidence of any oncologic advantages, the cosmetic benefits offered by robotic surgery are not enough to justify its extensive use in cancer patients. In contrast, the safety of this procedure can justify the use of the robotic technique in patient with benign/low grade malignant tumors of the pancreas.

The eighth paper describes the role of morphological and histological features of pancreatic stump in POPF occurrence after PD. Pancreatic texture, assessed by the surgeon, is a significant determining factor for pancreatic fistula and high grade pancreatic fistula and corresponds to pancreatic fibrosis grade. Moreover, careful consideration should be given to the larger pancreatic stumps, small Wirsung duct, wide pancreatic remnant mobilization, and duct decentralization on the stump anteroposterior axis. These morphological features also influence anastomosis failure.

Finally, the last paper discusses the efficacy of splanchnicectomy to relieve pain in pancreatic cancer. Transhiatal bilateral splanchnicectomy achieves a certain denervation of splanchnic nerves, but it requires a laparotomy. Unilateral thoracoscopic splanchnicectomy is a minimally invasive procedure to cause definite denervation. Bilateral thoracoscopic splanchnicectomy is recommended for unsatisfactory cases or recurrent pain occurring after an initial unilateral splanchnicectomy.

*Masahiko Hirota* *Juhani Sand* *Ralf Segersvärd* *Roberto Cirocchi*

